# Implantable sensor for local Cherenkov-excited luminescence imaging of tumor pO_2_ during radiotherapy

**DOI:** 10.1117/1.JBO.25.11.112704

**Published:** 2020-11-24

**Authors:** Xu Cao, Jason R. Gunn, Srinivasa Rao Allu, Petr Bruza, Shudong Jiang, Sergei A. Vinogradov, Brian W. Pogue

**Affiliations:** a Dartmouth College, Thayer School of Engineering, Hanover, New Hampshire, United States; b Ministry of Education, Xidian University, Engineering Research Center of Molecular and Neuroimaging, School of Life Science and Technology, Xi’an, Shaanxi, China; c University of Pennsylvania, Perelman School of Medicine, Department of Biochemistry and Biophysics, Philadelphia, Pennsylvania, United States; d University of Pennsylvania, School or Arts and Sciences, Department of Chemistry, Philadelphia, Pennsylvania, United States; e Norris Cotton Cancer Center, Dartmouth-Hitchcock Medical Center, Lebanon, New Hampshire, United States

**Keywords:** oxygen imaging, radiotherapy, Cherenkov, implantable probe, hypoxia

## Abstract

**Significance:** The necessity to use exogenous probes for optical oxygen measurements in radiotherapy poses challenges for clinical applications. Options for implantable probe biotechnology need to be improved to alleviate toxicity concerns in human use and facilitate translation to clinical trial use.

**Aim:** To develop an implantable oxygen sensor containing a phosphorescent oxygen probe such that the overall administered dose of the probe would be below the Federal Drug Administration (FDA)-prescribed microdose level, and the sensor would provide local high-intensity signal for longitudinal measurements of tissue ${\mathrm{pO}}_{2}$.

**Approach:** PtG4, an oxygen quenched dendritic molecule, was mixed into an agarose matrix at 100  μM concentration, allowing for local injection into tumors at the total dose of 10 nmol per animal, forming a gel at the site of injection. Cherenkov-excited luminescence imaging (CELI) was used to acquire the phosphorescence and provide intratumoral ${\mathrm{pO}}_{2}$.

**Results:** Although PtG4 does not form covalent bonds with agarose and gradually leaches out into the surrounding tissue, its retention time within the gel was sufficiently long to demonstrate the capability to measure intratumoral ${\mathrm{pO}}_{2}$ with the implantable gel sensors. The sensor’s performance was first evaluated *in vitro* in tissue simulation phantoms, and then the sensor was used to measure changes in oxygen in MDA-MB-231 tumors during hypofractionated radiotherapy.

**Conclusions:** Our study demonstrates that implantable oxygen sensors in combination with CELI present a promising approach for quantifying oxygen changes during the course of radiation therapy and thus for evaluating the tumor response to radiation. By improving the design of the gel–probe composition in order to prevent leaching of the probe into the tissue, biosensors can be created that should allow longitudinal oxygen measurements in tumors by means of CELI while using FDA-compliant microdose levels of the probe and thus lowering toxicity concerns.

## 1 Introduction

The effects of tumor oxygenation on the outcome of radiotherapy have been under investigation for decades.^1^^,^^2^ A multitude of studies have suggested that oxygen levels in tumors influence radiosensitivity of cells. Cancer cells in low-oxygen (hypoxic) environments can tolerate radiation doses 2 to 3 times higher than cells under normoxia.^3^^,^^4^ Oxygen enhances the radiobiologic damage through the action of oxygen-derived radical species, while hypoxia induces signaling cascades leading to adaptation and resistance.^5^^–^^7^ Oxygenation of tumors can serve as a prognostic factor for survival after radiotherapy, as patients with hypoxic tumors commonly exhibit poorer outcomes.^8^^,^^9^ Attempts have been made in numerous clinical trials to improve treatment outcomes by modifying oxygenation and/or the radiation dose delivered to tumors.^10^^–^^12^ To do that in an informed way, it is necessary to accurately quantify tumor oxygen levels, ideally periodically throughout the fractionation schedule. In this study, an approach to measuring oxygen during radiation delivery using small implantable sensors is examined that could be more easily deployed in clinics to aid radiotherapy verification and planning.

Polarographic electrodes have been the most common method to sample tumor partial pressure of oxygen (pO_2_) in experimental clinical trials.^13^ However, electrodes are invasive and cannot be easily translated to common clinical use. In recent years, imaging oxygenation with positron emission tomography (PET) and hypoxia-sensitive probes, such as fluoromisonidazole or fluoroazomycin arabinoside, has been evaluated in a number of studies.^14^^–^^16^ However, clinical use of PET is expensive, and imaging by PET must be scheduled as a separate procedure, which causes additional logistical difficulties. Furthermore, if used, PET is performed judiciously during the treatment course, typically providing only one snapshot of oxygenation at the time of initial treatment planning.^17^

Recently, we reported tumor ${\mathrm{pO}}_{2}$ measurements using Cherenkov-excited luminescence imaging (CELI) with a systemically delivered phosphorescent oxygen-sensitive probe PtG4.^18^^–^^20^ This technique makes use of the Cherenkov light generated within tissues subjected to radiation beams to excite the phosphorescence of PtG4, and the phosphorescence decay time of the probe is used to quantify tissue ${\mathrm{pO}}_{2}$. CELI offers several advantages, including real-time reporting of oxygenation in tumors at the time of delivering the radiation. However, so far this method has been used only in combination with systemic administration of PtG4. Systemic administration of any drug naturally requires extensive toxicological testing, including measurements of pharmacokinetics, biodistributions, and immunogenicity. Translating a probe into clinics would be significantly simplified if it could be delivered locally and remain in the delivery site without spreading throughout the body, thus eliminating the risks of global toxicity.

In this work, we evaluated the possibility of oxygen measurements with CELI using a model implantable sensor. The sensor was constructed by encapsulating PtG4 into the agarose gel. Originally, PtG4 was designed specifically for oxygen measurements in biological aqueous solutions.^21^^,^^22^ As with other dendritic oxygen probes,^23^ the molecule of PtG4 has a layer of polyethyleneglycol (PEG) residues at its periphery. The PEG layer makes PtG4 inert and incapable of forming chemical bonds with any encapsulating matrix, including agarose. However, because PtG4 is highly hydrophilic, it was simple to admix it to an agarose solution prior to gel formation, thus creating phosphorescent oxygen-sensitive gel sensors to conduct this proof-of-principle study. As expected, PtG4 leaches from the gel, and provided enough time, the gel becomes completely devoid of the probe. However, the retention time of PtG4 in the gel was found to be sufficiently long to allow local imaging of oxygen in tumors where the sensor with PtG4 was placed, and the release is local to the tissue of implantation anyway providing a localized delivery approach.

Our results show that using the local gel sensor delivery method, the overall dose of PtG4 per animal sufficient for CELI was ∼10  nmol. According to the Federal Drug Administration (FDA) guidelines, in animal model studies a microdose of an imaging agent should not exceed 30 nmol per mouse and must be at least 100 times less than the dose that might cause a pharmacological effect. Therefore, in terms of the quantities proscribed by the FDA, the agarose-PtG4 sensor already satisfied the requirements, and with further optimizations the dose levels can be reduced. Importantly, CELI with the agarose-PtG4 sensor provides reliable longitudinal oxygen readings from tumors at the time of radiotherapy, thus allowing radiation to be delivered at the most effective location and with optimal timing. The ability to perform ${\mathrm{pO}}_{2}$ sensing over multiple days in experimental tumors has also been confirmed.

## 2 Materials and Methods

### 2.1 Experimental Setup

The experimental setup for CELI has been detailed in the previous studies.^18^ A PI-MAX 4 1024i ICCD time-gated intensified camera (Princeton Instruments, Acton, Virginia) was used to collect luminescence excited by the radiation pulses provided by 6 megavolt (MV) x-ray beams, generated by a clinical linear accelerator (Varian LINAC 2100C, Varian Medical Systems, Palo Alto, California). The ICCD camera was coupled to a 135-mm f/2.0 lens (Canon) and fixed at a distance of ∼2  m from the imaging object. The x-ray beam was delivered by as 3.25  μs-long pulses at a 360-Hz repetition rate, and four luminescence images were acquired at different delay times after the pulse: 5, 10, 20, and 30  μs. The intensity of the phosphorescence in each image pixel was plotted as a function of the temporal delay, and the fit to an exponential function was used to determine the lifetime and calculate the local ${\mathrm{pO}}_{2}$.

### 2.2 Agarose-PtG4 Oxygen Sensor

**Fig. 1:**
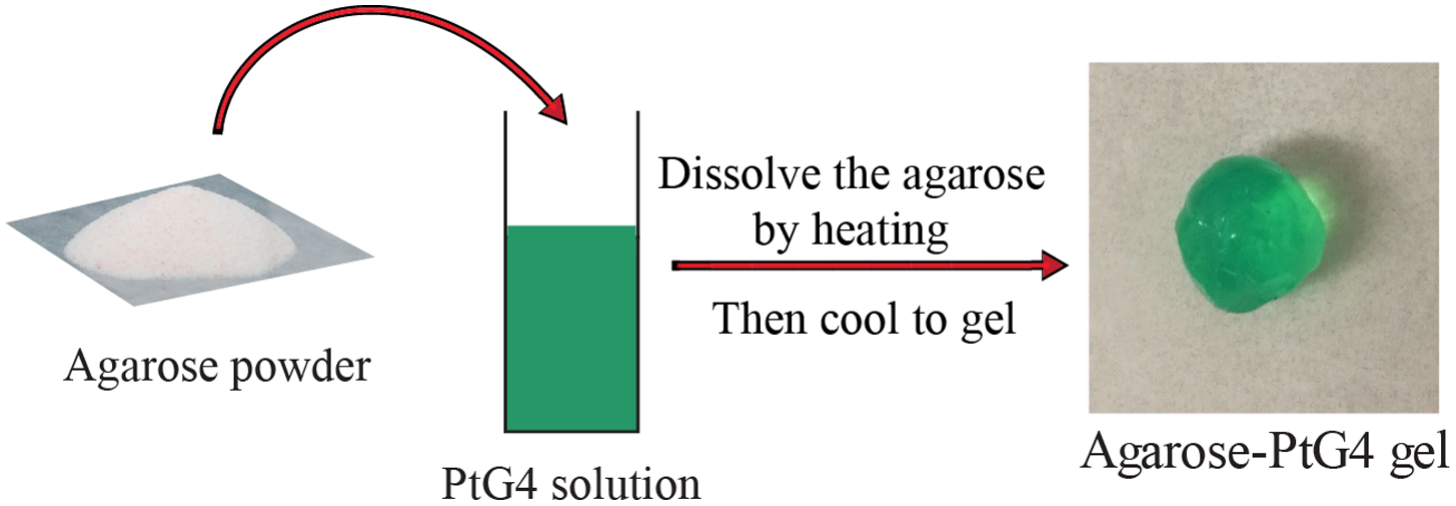
Fabrication of agarose-PtG4 oxygen sensor.

Agarose-PtG4 gel was employed as an implantable oxygen sensor. The fabrication process is illustrated in Fig. 1. The sensor was fabricated by mixing agarose with PtG4 solution at a stock concentration of 100  μM with an agarose concentration of 10 mg/ml. The agarose mixture was heated in a water bath to 80°C to 90°C to melt into a clear liquid. The gel formed upon cooling the mixture once the temperature was below $∼40^{{}^{\circ}}C$. Oxygen can easily diffuse into the agarose gel, and thus the agarose pellets retained oxygen sensitivity (see Fig. 4). When used at volumes of 0.1 ml, the subject dose was then 10 nmol, less than the required limit of 30 nmol for the FDA classification of microdosing.^24^^–^^26^

### 2.3 Phantom Experiments

For phantom studies, spherical sensor pellets of size $∼0.5\;{\mathrm{cm}}^{3}$ were fabricated. To test the response of the sensor to oxygen, a pellet was placed into a vial containing phosphate buffered saline (PBS), and oxygen was removed from the solution using the glucose/glucose oxidase/catalase system.^27^ Another vial was used as a control sample containing PBS with ambient dissolved oxygen level of ∼21%(${\mathrm{pO}}_{2}∼150\;\mathrm{mm}\mathrm{Hg}$). The two vials were imaged by CELI to verify the sensitivity of agarose-PtG4 gels to oxygen. Although the Stern–Volmer plot relating the phosphorescence decay time of PtG4 within the agarose gel to oxygen is different from the plot obtained for the probe dissolved freely in solution,^22^ for the purpose of our present experiments this difference was ignored, since it would affect only the absolute levels of ${\mathrm{pO}}_{2}$, but not the ability of the gel to sense oxygen. To investigate the possibility of imaging at depth, a vial with a sensor pellet kept at low oxygen was embedded into a tissue-emulating phantom, and imaging was performed as the sensor pellet was positioned at different depths in the medium. The phantom was made of PBS with added 1% Intralipid (soybean and egg yolk fat emulsion, Baxter Healthcare Corporation; Deerfield, Illinois), and 1% porcine blood (bovine whole blood in Na Heparin, Lampire Biological Laboratories; Pipersville, Pennsylvania) to match the scattering ($\mu_{s}\approx 10\;{\mathrm{cm}}^{-1}$) and absorption ($\mu_{a}\approx 0.1\;{\mathrm{cm}}^{-1}$) coefficients of soft tissue (muscle) at 800 nm.

### 2.4 *In Vivo* Experiments

**Fig. 2:**
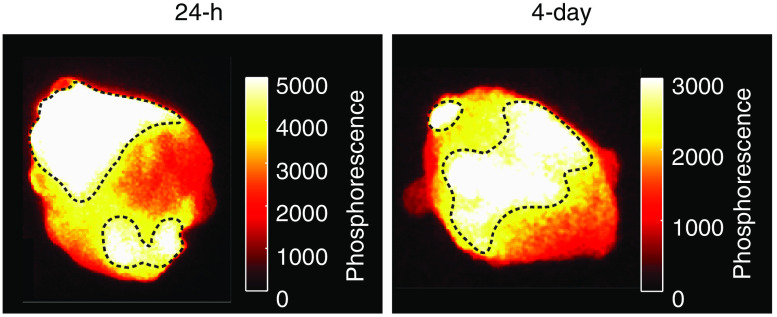
The agarose-PtG4 sensor inside the tumors at 24-h and 4-day time points. The regions of the sensor are marked by dotted lines.

All animal procedures were conducted following a protocol approved by the Dartmouth Institutional Animal Care and Use Committee. Nude female mice 6 to 8 weeks of age (Charles River Labs, Wilmington, Massachusetts) were involved in this study. $10^{6}$ MDA-MB-231 tumor cells (purchased from PerkinElmer/Caliper with Cat. No.: 119369) were injected subcutaneously under the skin on the flank of each mouse. After ∼2weeks of growth, animals were chosen for imaging when their tumor diameter reached ∼8  mm in size. 100  μL of the agarose mixture was injected directly into the tumor when the solution was still a thick liquid ($∼40^{{}^{\circ}}C$). The mixture solidified into the gel inside the tumor after 1 to 3 min. The formation of the gel in the tissue was confirmed by excising the tumors and examining them visually as well as by imaging phosphorescence (Fig. 2). The tumors contained agarose-PtG4 sensors in them outlined by the dashed line in Fig. 2 based on its much stronger phosphorescence intensity comparing to that of the diffused areas, notwithstanding that some probe leaked into the surrounding tissue. CELI was carried out during the fractionated radiotherapy treatment to the tumor while the animals were kept under anesthesia (isofluorane 1% to 3% admixed to $O_{2}$).

## 3 Results

### 3.1 PtG4 Leaching from Agarose-PtG4 Sensor

**Fig. 3:**
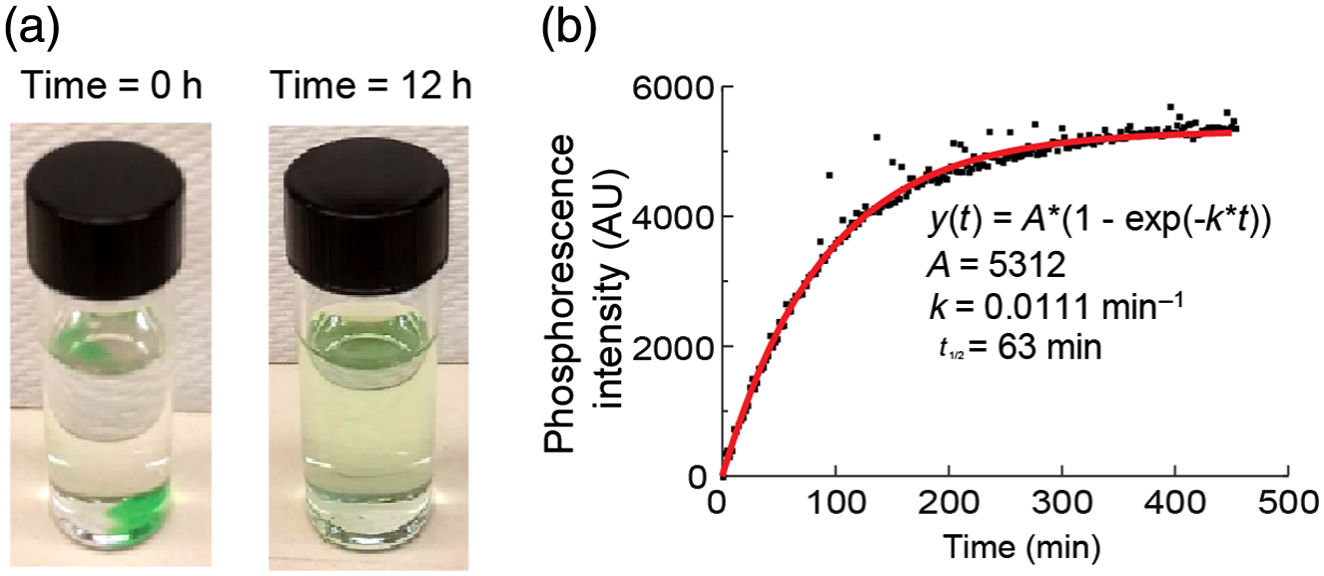
PtG4 leaching from agarose-PtG4 sensor in water. (a) Agarose-PtG4 sensor in a vial with water immediately after immersion and 12 h later. (b) Quantification of PtG4 leaching: increase in the phosphorescence intensity from solution over time.

First, we evaluated the rate of leaching of PtG4 from the agarose gel into the surrounding solution (Fig. 3). Initially, the color of the agarose-PtG4 pellet immersed into water was deep green, but gradually the pellet faded and after ∼12  h completely lost color as PtG4 leached into solution [Fig. 3(a)]. The leaching was accompanied by an increase in the phosphorescence intensity measured from the solution [Fig. 3(b)]. The half-time of PtG4 leach was found to be ∼1  h.

### 3.2 Oxygen Sensitivity of the Agarose-PtG4 Sensor

**Fig. 4:**
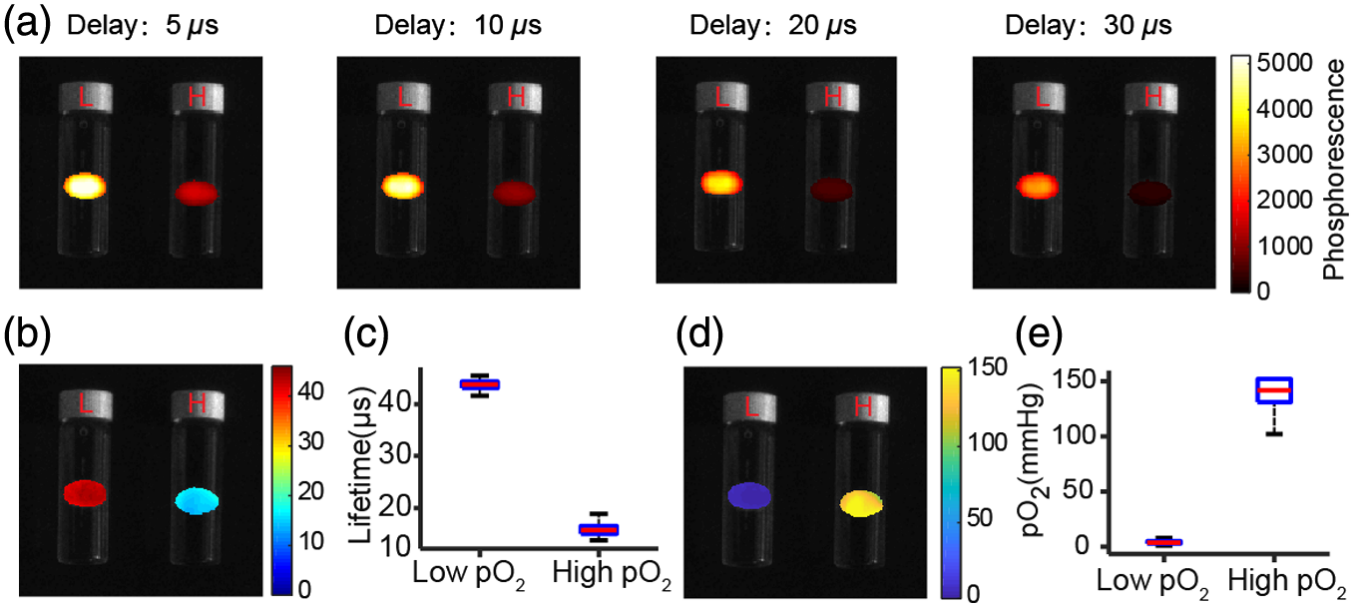
Oxygen sensitivity of the agarose-PtG4 sensor in aqueous environment. (a) Phosphorescence images acquired at different time delays after 4-μs-long radiation pulses. Sensor pellets were sealed in two vials containing PBS. The corresponding (b) lifetime images, (c) quantitative lifetime values, (d) calculated oxygen images, and (e) quantitative ${\mathrm{pO}}_{2}$ values, respectively. Boxplots show median and interquartile range; whiskers indicate the range. “L” and “H” labels on the vials indicate the low- and high-oxygen environments, respectively.

The CELI images of agarose-PtG4 sensor pellets in high- and low-oxygen environments were acquired at different delay times as shown in Fig. 4(a). As expected, the phosphorescence intensity of the sensor at high oxygen was lower than that at low oxygen. This confirmed that oxygen from solution can diffuse into the gel and quench the phosphorescence of the PtG4 in the gel. The lifetime maps were obtained by exponential fitting of the phosphorescence signals versus delay time in each pixel [Fig. 4(b)]. The median lifetimes over all the pixels of agarose-PtG4 sensor in high- and low-oxygen environments were 15 and 45  μs, respectively [Fig. 4(c)]. The ${\mathrm{pO}}_{2}$ images of the sensor pellets were obtained by applying the Stern–Volmer calibration of the probe in solution [Fig. 4(d)]. Briefly, an OxyLED phosphorometer (Oxygen Enterprises Ltd.) and an oxygen electrode were used to measure the ${\mathrm{pO}}_{2}$ of the PtG4 solution at the same time. A cylindrical vial with PtG4 solution was fitted with a silicon rubber stopper, and two stainless steel needles were inserted into the vial serving as gas inlet and outlet. Argon was bubbled into the vial to gradually replace oxygen and other dissolved gases, phosphorescence lifetimes and ${\mathrm{pO}}_{2}$ were synchronously recorded for the subsequent Stern–Volmer analysis.^22^ Since the calibration of PtG4 in the gel is different from that in water, the ${\mathrm{pO}}_{2}$ values reflect oxygen in the environment only approximately. The approximate median ${\mathrm{pO}}_{2}$ values are shown in Fig. 4(e).

### 3.3 Imaging Depth of CELI

**Fig. 5:**
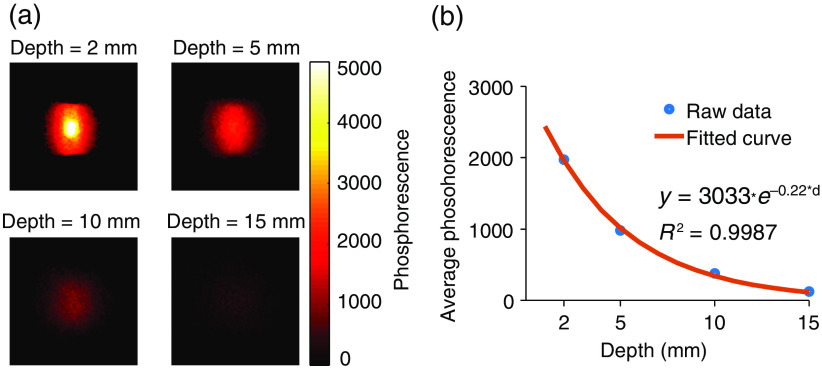
Imaging depth of CELI with sensor in tissue phantom. (a) Phosphorescence images acquired at different depths in liquid diffuse tissue-simulating phantom medium. (b) Phosphorescence signal intensity decayed exponentially with depth into the tissue-like medium.

Phosphorescence images of the sensor pellets at different depths in liquid tissue-simulating media were used to investigate the potential to carry out oxygen imaging at depth [Fig. 5(a)]. Quantification of phosphorescence intensity showed an approximately exponential decay [Fig. 5(b)] with the constant of $0.22\;{\mathrm{mm}}^{-1}$, corresponding to the expected effective attenuation coefficient of this medium in the near-infrared spectral region. The maximal imaging depth was more than 10 mm, which is sufficient to measure ${\mathrm{pO}}_{2}$ in superficial tumors in humans such as some soft tissue sarcomas

### 3.4 *In Vivo* Oxygen Sensing

**Fig. 6:**
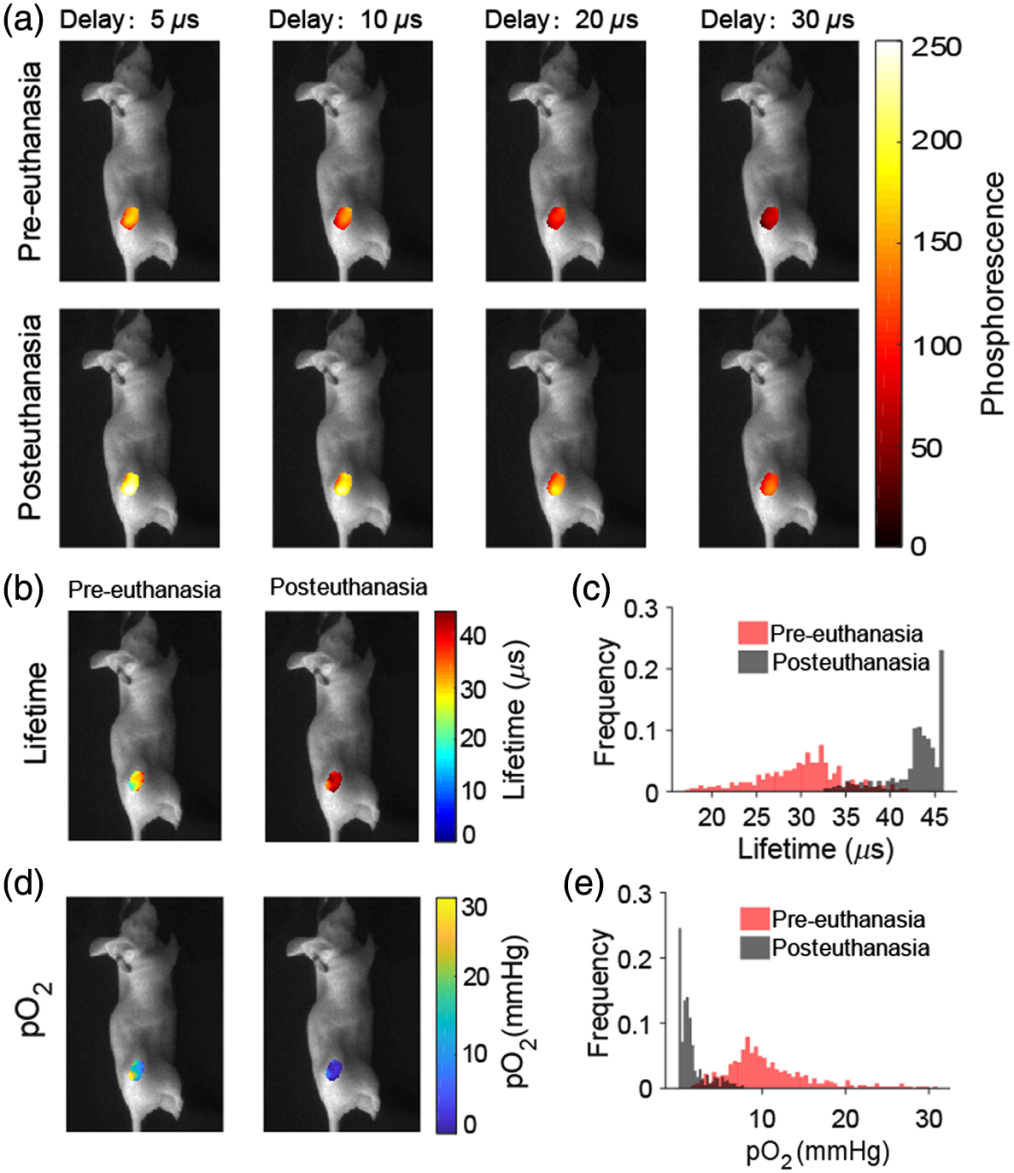
Tumor oxygen imaging before and after euthanasia. (a) Phosphorescence sensor images acquired at different time delays for pre-euthanasia and posteuthanasia. (b) Lifetime images and (c) corresponding histograms. (d) ${\mathrm{pO}}_{2}$ images and (e) corresponding histograms.

To test the ability to sense oxygen in living tissue, the gel sensors were implanted into subcutaneous MD-MBA-231 tumors at the flank of the mice. Phosphorescence images at different delay times were acquired before and 30 min after sacrificing the mice by cervical dislocation [Fig. 6(a)]. Phosphorescence signals in posteuthanasia tumor were higher than those in the pre-euthanasia tumors. The lifetime maps were obtained by fitting pixel-by-pixel the intensity images with single-exponential functions [Fig. 6(b)]. The distributions of the lifetimes in pre-euthanasia versus posteuthanasia tumors were different as shown in Fig. 6(c). Then oxygen maps were calculated using the probe calibrations in solution as opposed to the gel. Therefore, the ${\mathrm{pO}}_{2}$ distributions shown do not reflect the actual ${\mathrm{pO}}_{2}$ values, but qualitatively show areas of higher and lower oxygenation [Fig. 6(d)]. The ${\mathrm{pO}}_{2}$ values in pre-euthanasia tumor were higher than in posteuthanasia, as shown in the ${\mathrm{pO}}_{2}$ histograms [Fig. 6(e)].

**Fig. 7:**
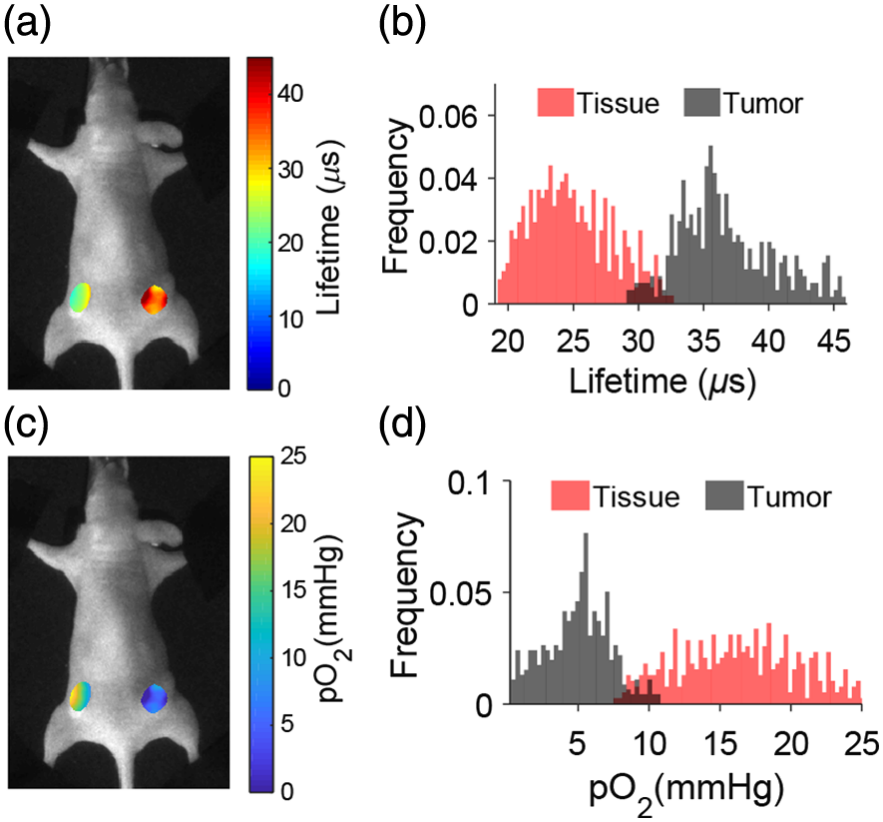
Qualitative oxygen imaging for tumor and muscle. (a) Lifetime images of tumor and muscle tissues with (b) histograms. The corresponding (c) oxygen images and (d) histograms.

Oxygen levels in subcutaneous MD-MBA-231 tumors and in normal tissues were further investigated by CELI using the implanted sensors. The lifetime maps for a tumor and normal tissue (muscle) are shown in Fig. 7 along with the histograms and the qualitative oxygen maps.

### 3.5 Sensing of Oxygen during Radiotherapy

**Fig. 8:**
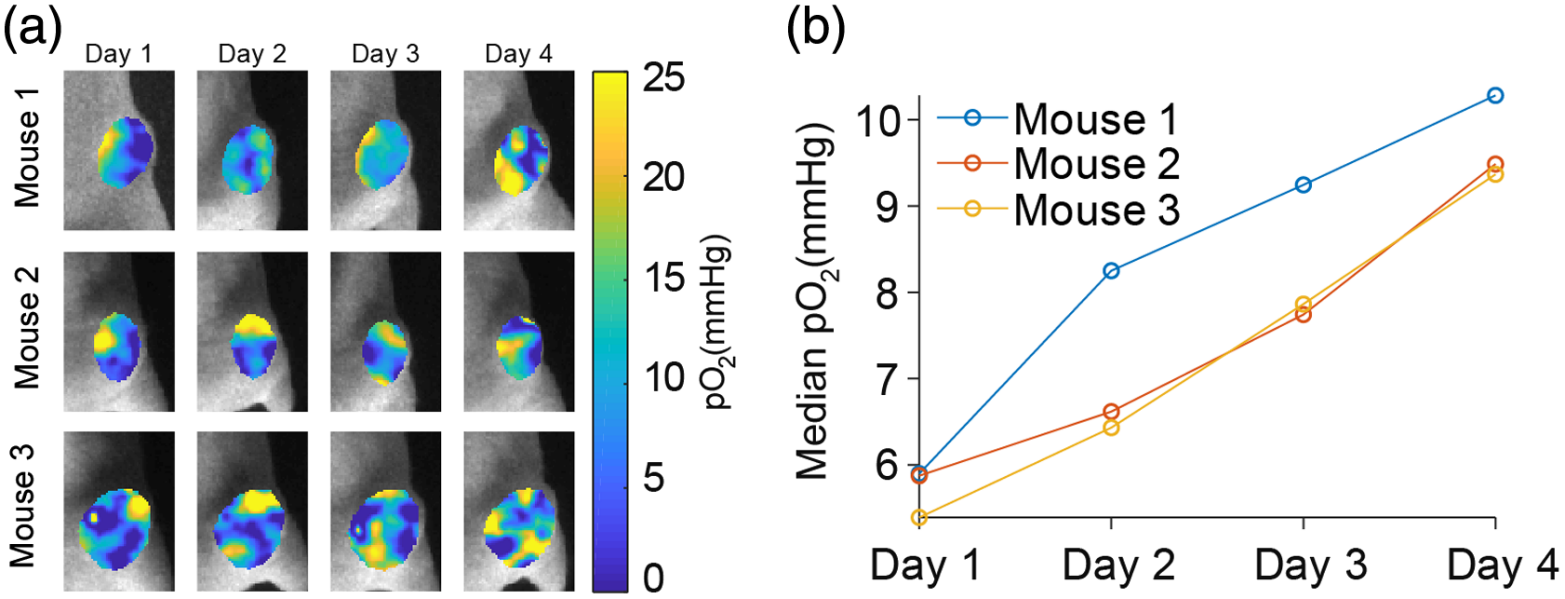
Tumor oxygen sensing during 4 days of fractionated radiotherapy. (a) Oxygen images in tumors in three mice taken during radiation treatment (5 Gy dose). (b) Median ${\mathrm{pO}}_{2}$ values as a function of time.

To monitor oxygen changes in tumors undergoing multiple fractions of radiotherapy, gel sensors were implanted into three mice with subcutaneous MDA-MB-231 tumors on day 1 and imaged by CELI during each fraction of radiotherapy. A 6 MV photon beam was delivered to each tumor with 5 Gy/fraction for 4 daily fractions, for a total of 20 Gy. The ${\mathrm{pO}}_{2}$ maps of MDA-MB-231 tumors indicated that the spatial ${\mathrm{pO}}_{2}$ distributions varied with time during the progression of radiotherapy [Fig. 8(a)]. The median ${\mathrm{pO}}_{2}$ values within the tumors increased with the progression of the radiotherapy [Fig. 8(b)].

## 4 Discussion

The ability to assess ${\mathrm{pO}}_{2}$ during radiotherapy would be invaluable for planning and optimization of treatment protocols. Translation of diagnostic tools, such as CELI, into clinical use could help to enable measurement of tissue ${\mathrm{pO}}_{2}$ during each fraction of radiation delivery. Here, we used phosphorescent probe PtG4 embedded into the agarose gel to sense tumor ${\mathrm{pO}}_{2}$ during radiation treatment by means of CELI. This technology has the advantage of being able to quantify tumor ${\mathrm{pO}}_{2}$ locally and reveal regions of hypoxia during each radiotherapy session, while avoiding the necessity to deliver the probe systemically.

PtG4 has been designed to sense oxygen in aqueous solutions, and its accumulation in tissue is governed by extravascular diffusion and retention kinetics. The toxicology, inflammatory, and immunogenicity properties of PtG4 have not been fully elucidated, so it cannot be translated to clinic at present, although this could be achieved in the future. However, the delivery of an oxygen-sensing probe encapsulated in a gel, e.g., by a direct intratumoral injection, should allow local oxygen sensing, while avoiding systemic circulation and associated toxicity issues. Local injection is a well-known way to provide microdose levels to a tissue region. Sufficiently long retention time of PtG4 within the agarose gel allowed us to perform ${\mathrm{pO}}_{2}$ imaging over several days, as the probe that leached out from the gel pellet remained for the most part within the surrounding tumor tissue for local delivery.

Almost all optical imaging technologies are critically limited by the depth at which they can sense in tissue. If an optical method is used in clinical oxygen monitoring, the tissue absorption and scattering characteristics are the factors that limit the depth of imaging the most. One advantage of CELI is that Cherenkov emission is produced within the tissue, providing an inner excitation source generated along the pathways of the MV x-ray beams. Thus, PtG4 can be excited at all depths where the radiation dose is deposited. The only major limitation is the modest intensity of Cherenkov emission, which results in a very low-phosphorescence signal. The biosensor produced in this study was directly injected into tumor, and that allowed us to effectively enhance the phosphorescent signals by increasing local concentration of PtG4 inside the tumor.

Clinical imaging of ${\mathrm{pO}}_{2}$ in near-surface tumors, such as head and neck tumors, by CELI should become possible if gel-based sensors are created that contain covalently bound, nonleaching probes, which would alleviate toxicity issues. The leaching rate of PtG4 into external solution was sufficiently low ($t_{1/2}∼1\;h$) to evaluate the response of the probe captured inside the agarose-PtG4 gel to oxygen as well as to image phosphorescence from the agarose-PtG4 gel implanted into tissue. The porous structure of the gel facilitated oxygen penetration, but the high leaching rate precluded truly quantitative longitudinal *in vivo* studies. Nevertheless, our experiments suggest that with proper chemical design, gel-based materials may be useful as local probes for CELI of oxygen. And in some cases, gels that are able to release the probe into the nearby tissue may in fact be advantageous for sensing over a larger tumor volume. In this regard, agarose-PtG4 gel pellets described in this study will be further evaluated as local probe delivery vehicles.

## 5 Conclusions

In summary, an implantable oxygen sensor has been developed for CELI of oxygen by encapsulating water-soluble phosphorescent probe PtG4 into agarose gel. The agarose-PtG4 solution could be injected into the tumor prior to gelation, which provided an opportunity to monitor tumor oxygen pressure during radiotherapy. This type of sensor design and delivery could significantly decrease concerns related to the toxicity of oxygen probes in clinical setting. The approach overall can provide a method for monitoring tumor hypoxia in clinical radiotherapy and thus help to identify hypoxic areas in tumors for planning and optimizing therapeutic intervention.

## Code, Data, and Materials Availability

The data that support the plots and figures within this paper and other findings of this study are available from the corresponding author upon reasonable request.
